# Comparison of Recycled Polymers with Their Virgin Counterparts: Properties and Eco-Indicators

**DOI:** 10.3390/polym18182185

**Published:** 2026-09-08

**Authors:** Carmen Alonso Herr, Marina P. Arrieta

**Affiliations:** 1 Departamento de Ingeniería Química Industrial y del Medio Ambiente, Escuela Técnica Superior de Ingenieros Industriales, Universidad Politécnica de Madrid ETSII-UPM, C/José Gutiérrez Abascal 2, 28006 Madrid, Spain; 2 Grupo de Polímeros, Caracterización y Aplicaciones (POLCA), C/José Gutiérrez Abascal 2, 28006 Madrid, Spain; 3 CIME, Centro de Investigación en Materiales Estructurales de la Universidad Politécnica de Madrid, C/Profesor Aranguren 3, 28040 Madrid, Spain

**Keywords:** recycling, plastics, polymers, circular economy, recycled plastics, sustainability

## Abstract

Plastics represent one of the biggest challenges related to the circular economy (CE) and sustainable development goals (SDGs). In this study, both the virgin and mechanically recycled versions of three traditional petroleum-based polymers, polyethylene (PE), polypropylene (PP) and polyethylene terephthalate (PET), are compared. For that, an investigation of the different mechanical, chemical and thermal properties was conducted. Additionally, the life cycle eco-indicators were studied for each case. Finally, correlations between all of the factors were studied in order to get a more comprehensive understanding of how the properties influence one another.

## 1. Introduction

There is an urge to find a sustainable solution in this field, and recycling polymers seems to be one of the most promising options. In line with the sustainable development goals (SDGs), the European Union has strengthened its legislative framework through the Packaging and Packaging Waste Regulation (PPWR) [1], which promotes a circular economy by increasing packaging recyclability, reducing packaging waste, and encouraging the incorporation of recycled materials into new packaging materials. These measures aim to achieve the recycling targets of a 65% overall recycling rate for packaging waste by the end of 2025, increasing to 70% by 2030, as previously established under the European Directive 94/62/EC [2], as amended in 2018, and a plastic packaging recycling rate of at least 50% by 2025 and 55% by 2030. Thus, investing in the plastic packaging sector is not an suggestion but a requirement. The aim is to focus on increasing the recyclability rate which is currently 38% in the EU, according to Eurostat, increase the ratio of recyclates in packaging and support reusability [3].

Only 9% of plastic waste is recycled, 15% is collected for recycling, 50% ends up in landfill, and 22% ends up in a waste stream to soil or water [4]. During the mechanical recycling process, the materials are subjected to heat and mechanical stress. Consequently, the recycled materials would not, in general terms, possess the same properties as their virgin counterparts.

With scientific advances, only 2% of materials can maintain the required mechanical, barrier and optical properties. The rest will exhibit lower properties than those necessary for packaging [3].

Another problem is the recyclability rate. The recyclability in manufacturing varies depending on the properties. This means that the supply chain could slow down, depending on the polymer. In this regard, PET (19.5%) is the polymer with the highest recyclability rate, followed by high-density polyethylene (HDPE, 10.3%), low-density polyethylene (LDPE, 5.30%), PS (0.9%) and PP (0.6%) [5].

Among all traditional petrochemical-derived polymers, PET is currently the only post-consumer recycled packaging polymer with a well-established closed-loop recycling infrastructure, such that it enables recycled PET (RPET) to be incorporated into new food-contact packaging in alignment with the circularity objectives promoted by the PPWR, primarily through bottle-to-bottle recycling [6]. PET has a much larger environmental footprint than the other polymers in terms of eutrophication, human health, ozone depletion and acidification. This is because it has the highest environmental impact, mainly due to high human-toxicity potential, as compared to polyolefins such as PE and PP. When comparing virgin and recycled materials, the carbon footprints are as follows: PET (virgin 2.23, recycled 0.91); PE (virgin 1.89, recycled 0.56); and PP (virgin 1.84, recycled 0.53). The environmental impact of treatment is positive due to the environmental benefits of the products avoided [7]. For instance, when using PET as an example, PET recycling reduces energy consumption by 84% and greenhouse gas emissions (GHGs) by 71% which is significantly more than virgin PET production [8].

The plastics studied in the present research are PE, PP and PET. PE and PP are polyolefins that together account for approximately 50% of the market, whereas PET is a polyester produced by polycondensation which represents less than 10% of the market; recycling rates of PET are considerably higher than for polyolefins, mainly through the mechanical recycling process, particularly for PET beverage bottles [9].

After the mechanical recycling process, due to reprocessing, plastics turn grayish and lose their brightness because small amounts of residues or degradation products remain. Their toughness also increases, but this can be compensated by increasing the weight, which has to be taken into account in logistics, transportation and manufacturing [10]. Also, it must be taken into account that there is only a limited number of times that plastic can be recycled so that the mechanical properties can be more or less maintained. This is due to crystallinity, fatigue resistance, molecular weight reduction and Young’s modulus decrease [11]. In order to prolong the life cycle, additives and composites are commonly used, improving or maintaining the mechanical properties. For instance, when comparing recycled PP with its virgin counterpart, it was shown that the properties of the recycled material are a little bit lower due to its service life and heat processing stage [12]. In this study, it was found that both the virgin polymer and the recycled one formed alpha and beta crystals, although the recyclated PP showed a higher proportion of beta crystals, which were associated with a lower Young’s modulus.

Changes were observed in a comparative study of polyethylene [10], and an increase in the crystallization temperature was found, which indicates that the crystallization rate increases, as in the previous study. It may be because of reprocessing or a second extrusion cycle; no significant difference was observed in either the tensile strength or permeability. As for PET, the Young’s modulus of the recycled PET (RPET) samples is greater than the Young’s modulus of the virgin PET, indicating a higher level of stiffness or rigidity, due to its greater density [13].

Therefore, the current work aims to analyze the relationship between eco-indicators and the physico-chemical and mechanical properties of commodity plastics, specifically virgin and recycled PET, PP and PE, in order to assess the influence of the mechanical recycling process on their environmental and functional performance.

## 2. Materials and Methods

### 2.1. Materials

The materials used were polyethylene terephthalate (PET), polypropylene (PP), polyethylene (PE) and their recycled counterparts RPET, RPP and RPE. Materials mechanically recycled for the first time (1 cycle) were obtained from European companies whose identities cannot be disclosed; it can be said that the same company provided the virgin and recycled polymers.

### 2.2. Methods

#### 2.2.1. Attenuated Total Reflectance Fourier Transform Infrared Spectroscopy

The functional groups and molecular interactions were assessed using a Jasco FT/IR-4X spectrometer (Jasco, Euston, MD, USA) equipped with an ATR (attenuated total reflectance) accessory. The spectra used a spectra manager 2005 software with 64 scans in the range 400–4000 cm^−1^ at room temperature with a resolution of 4 cm^−1^.

#### 2.2.2. Field Emission Scanning Electron Microscopy

The cross section of the materials was analyzed from samples previously subjected to cryo-fracture cutting using liquid nitrogen. They were observed by field emission scanning electron microscopy (FESEM) using a JEOL JSM-IT700HR (Oxford Instruments, Tokyo, Japan). The samples were accelerated at a voltage of 15 kV with magnifications of 10 and 50.

#### 2.2.3. Tensile Test

Tensile test measurements were conducted using a Shimadzu AGS-X-100N (Kyoto, Japan) equipped with a 100N cell operated via TRAPEZIUM X software (version 1 5.4). The initial separation was 30 mm, and the crosshead speed was set to 10 mm/min in a dog-bone shape specimens (specimen type 1BB) according to ISO 527:2025 [14] and following the general principles established in ISO 527-1:2019 [15]. Young’s modulus (E), tensile strength and elongation at break were calculated from the resulting stress and strain curves. In this case, the experiment was repeated 5 times per sample (*n* = 5).

#### 2.2.4. Water Vapor Transmission Rate (WVTR)

The rate of water vapor transmission through the films was determined following UNE 53097 [16], which determines a gravimetric method for evaluating water-transferring materials using silica gel as a desiccant agent. Samples were placed in permeability cups filled with 2 g of dried silica. Each capsule, with an exposed surface area (*A*) of 10 cm^2^, was weighted every hour for the first 6 h and then again at 24 h until the weight gain stabilized in order to plot the weight gain as a function of time (*t*). Each experiment was repeated 3 times (*n* = 3). The slope of the curve (*a*) of the linear portion was used to calculate *WVTR* in g/day.m^2^ and then normalized to a film thickness (*e*) of 100 µm.

$$WVTRnorm=\frac{24\;a\;e}{A\;t\;0.1}$$

#### 2.2.5. Thermal Properties

The thermal stability of the materials was analyzed by thermogravimetric analysis (TGA), measuring the loss of weight as a result of heat, which also gives some hints on the chemical structure of the molecules. The TGA used was a SETARAM (Caluire, France), and the experiments were conducted under a N_2_ atmosphere.

Differential scanning calorimetry analysis (DSC) was conducted on a SETLINE DSC SETARAM under a nitrogen atmosphere. The first and second heating scans were conducted from −60 °C to 300 °C at 10°C/min. Then the samples were cooled down at the same rate.

#### 2.2.6. Life Cycle Assessment

Life cycle analysis is a useful tool that follows the path that a material creates between the extraction until the end of the cycle. In this case, “Cradle to grave” will be studied—analyzing all the entire life cycle from production to disposal.

The life cycle is a methodology that evaluates the environmental sustainability of the process and some systems throughout their life cycle. It is standardized [17,18], and it is defined in the following stages: (1) aim; (2) life cycle inventory; (3) evaluation of the impact of life cycle; (4) interpretation. It can be detailed below:

Step 1: Inventory of life cycle

The scope of the project is limited to the entry and exit flow with the study system. It encompasses the gathering and quantification of all the necessary data in order to meet the targets.

Step 2: Life cycle analysis

The analysis takes into account the environmental calculus of the environmental impact of all of the inputs and outputs. For the analysis, OpenLCA (version 2.7.0, GreenDelta GmbH, Berlin, Germany) and SimaPro (version 9.6, PRé Sustainability B.V., Amersfoort, The Netherlands) had been used, as well as the PEF (product environmental footprint) as a data baseline. This was created by the European Commission (Joint Research Centre). With all of this, the following eco-indicators were calculated: acidification, ecotoxicity, eutrophication, ionizing radiation, land use, and ozone depletion.

Step 3: Interpretation

In the last phase, all of the results of the inventory and analysis are discussed and used as a baseline for conclusions and recommendations.

## 3. Results

**Figure 1:**
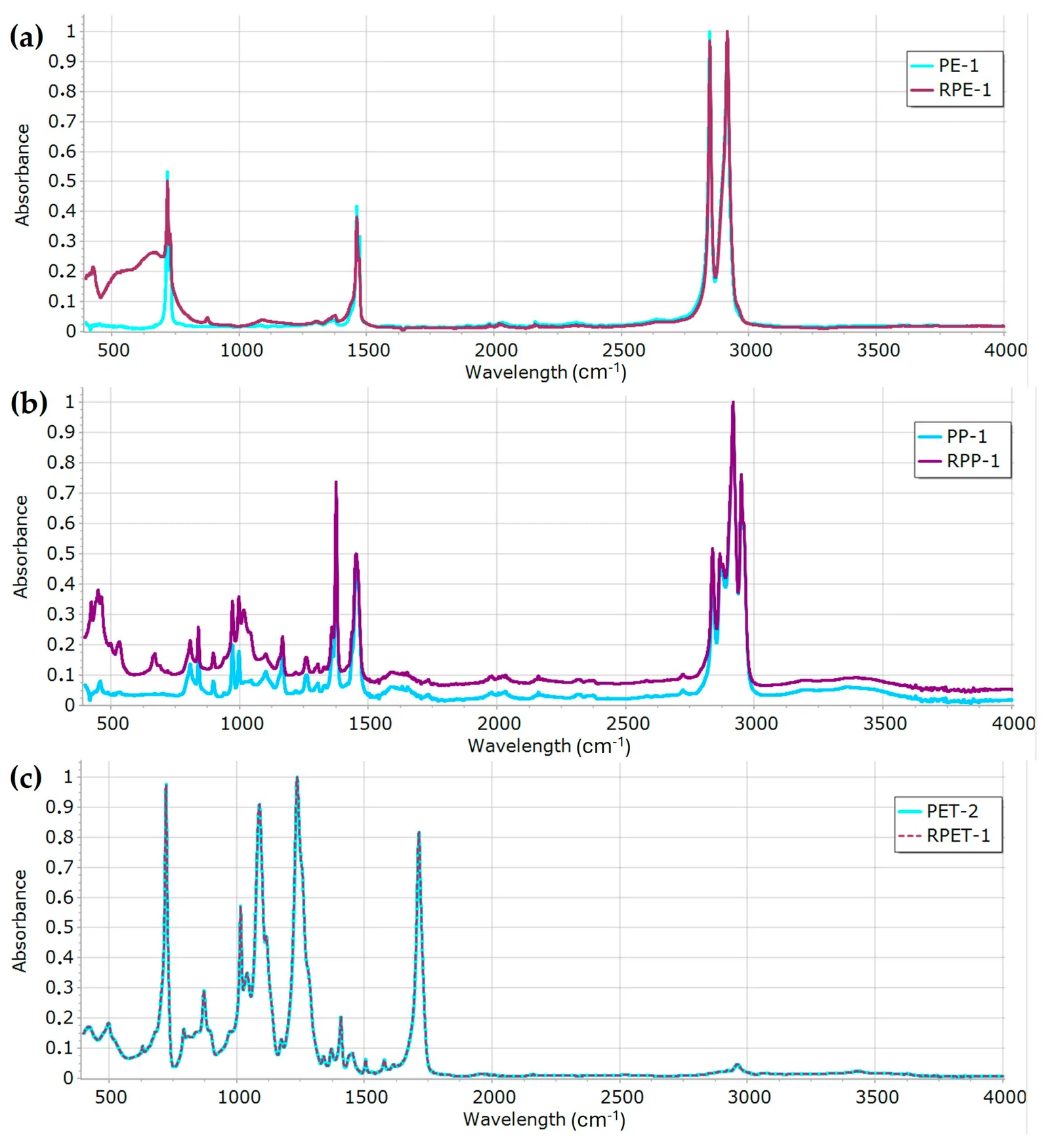
ATR-FTIR spectra of: (**a**) PE and RPE; (**b**) PP and RPP; (**c**) PET and RPET.

In Figure 1a, the FTIR spectra of PE and rPE are shown. There is a slight area increase from 400 to 700 cm^−1^, which may be due to the addition of additives in order to improve the materials’ properties. PE has a rocking band at 700 cm^−1^, which is much broader in the recycled material due to the loss of crystallinity [19]. The major peak occurs at 2800–2900 cm^−1^ which corresponds to the stretching vibration of the -CH_2_ (methylene) group and at 1450 cm^−1^ for the bending deformation (scissoring) of the -CH_2_ group. In the same way as with polyethylene, the recycled PP polymer shows a great peak in the 400–1200 cm^−1^ region (Figure 1b). In general, the characteristic peaks show more intensity. The region from 2850 to 3000 cm^−1^ corresponds to asymmetric and symmetric stretching of methyl (-CH_3_) groups. At 1375 cm^−1^, the main peak shows the symmetric bending vibration of the methyl (-CH_3_) group, differentiating it from PE. The absorption spectra of PET and RPET are very similar. PET has characteristic peaks due to its bond with oxygen, including the following: 1750 cm^−1^: strong stretching vibration of the ester carbonyl group (C=O); 1240 cm^−1^: asymmetric stretching of the ester group or aromatic-aliphatic ether bond (C-O); 1100 cm^−1^: stretching vibration of the methylene group/aliphatic ether (C-O) or ester backbone. The very similar ATR-FTIR spectra of PET and RPET suggest that no major chemical changes in the characteristic PET absorption bands were detected, with no major degradation during recycling [10].

**Figure 2:**
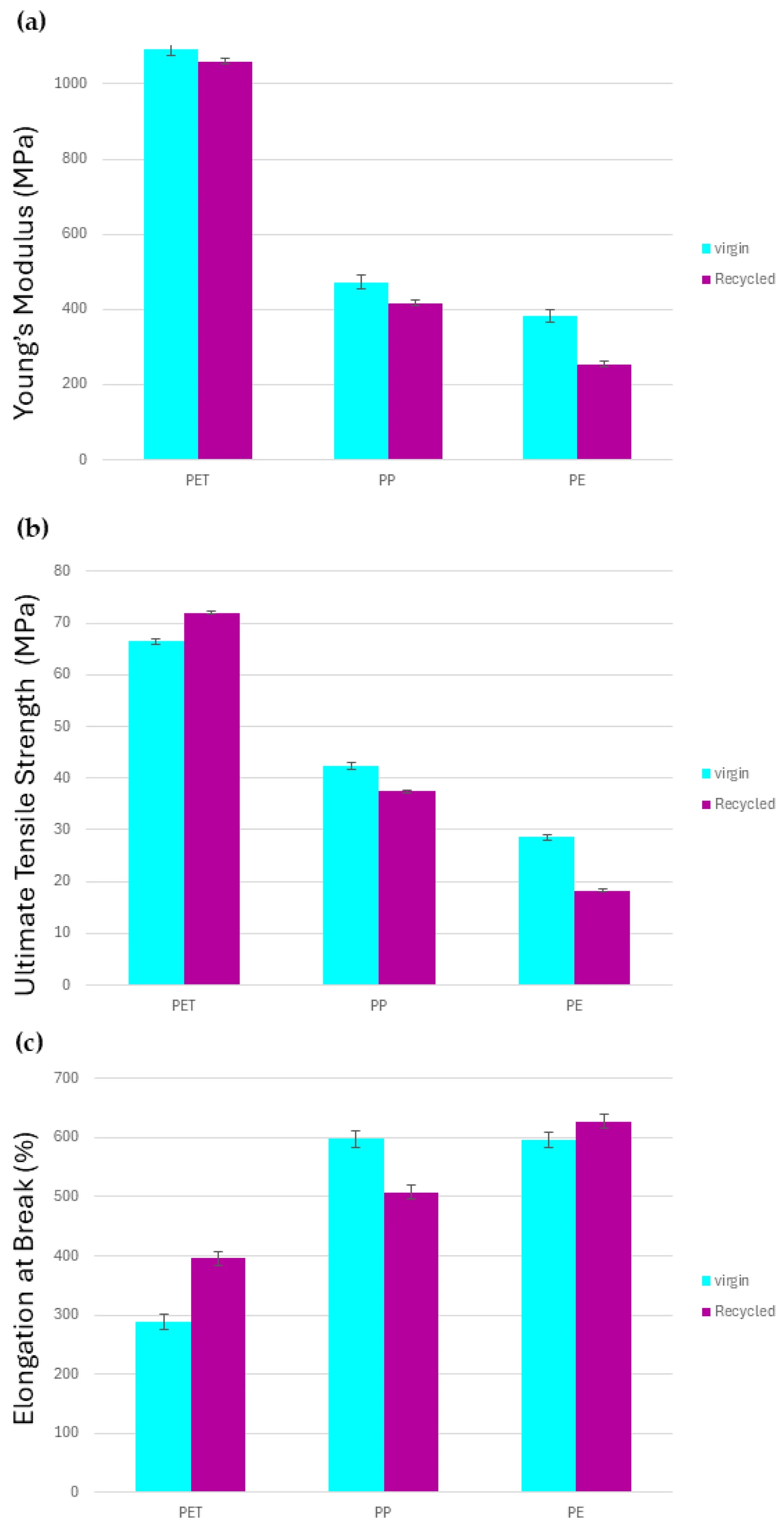
Tensile test properties of virgin PET, PP, PE and recycled counterparts: (**a**) Young’s modulus; (**b**) ultimate tensile strength; and (**c**) elongation at break.

The mechanical properties of the virgin and recycled materials were studied by tensile test measurements. As Figure 2 shows, PE has low intermolecular forces and long elongation; PP has greater rigidity but still chain mobility; and PET has less extreme elongation. In the case of recycled materials, oxygen attacks the amorphous phase due to the higher mobility of the chains, as it is more difficult to interact with the crystalline domain, which leads to more degradation [20]. PET has great rigidity (high E and UTS, and low elongation at break) due to the periodic organization in crystalline regions separated by an amorphous phase and the rigid ester groups [13]. Also, it can be noted that in both cases, the values of the recycled materials are similar to the virgin counterparts, which implies the same chemical bonds and structure. The elongation of the recycled is greater, which may be due to weaker bonds or more entanglement, whereas the virgin material is more structured, with higher fractions of crystallinity. As for the elongation, it rapidly reduces due to thermo-oxidative degradation, which causes scission of the chains [21].

PP is more prone to chain scission, which makes it brittle, depending on the processing method. The mechanical properties of the polypropylene depend on the stress the polymer suffers during each extrusion process and the crystallinity of the polymer. The higher the crystalline rate of the polypropylene, the less the polymer will elongate. The mechanical recycling of the polymer may induce chain scission and changes in molecular weight and crystallinity domains, so it can influence the tensile behavior [22].

For PE, the recyclate has a significantly lower UTS, which may be due to contaminants introduced during the manufacturing process as opposed to the number of cycles [23]. PE is prone to crosslinking and chain branching during mechanical recycling, increasing the entanglement of the chains so that the elongation remains. The E is lower than virgin PE, which can be due to the distribution of phases [24].

The water vapor transmission rate (WVTR) results of virgin and recycled polymers are shown in Table 1. Increased molecular distance results in increased facility of the water when passing through [10]. As mentioned before, PP causes chain scission, which reduces the molecular weight and modifies its physical–mechanical properties; it also adds up polymers in its free volume. This affects PP and RPP, as when the free volume increases, the permeability does too [25]. This suggests that there is an increase in polymer chain mobility. WVTR of PET stays unchanged. Even though during reprocessing chains are reduced, there is an increase in PET molecular weight by chain extension reactions [26]. In PE, mechanical recycling may induce changes in the molecular architecture through chain scission, which can be destroyed and united in star-like and comb-shaped chains rather than long-chain branching attributed to antioxidant depletion [27,28], which increases the WVTR due to the increase in the mobility of the chains. In this work, it seems that the mechanical recycling of PE increased the permeability from 0.08 g/day m^2^ in PE to 0.34 g/day m^2^ in RPE.

**Figure 3:**
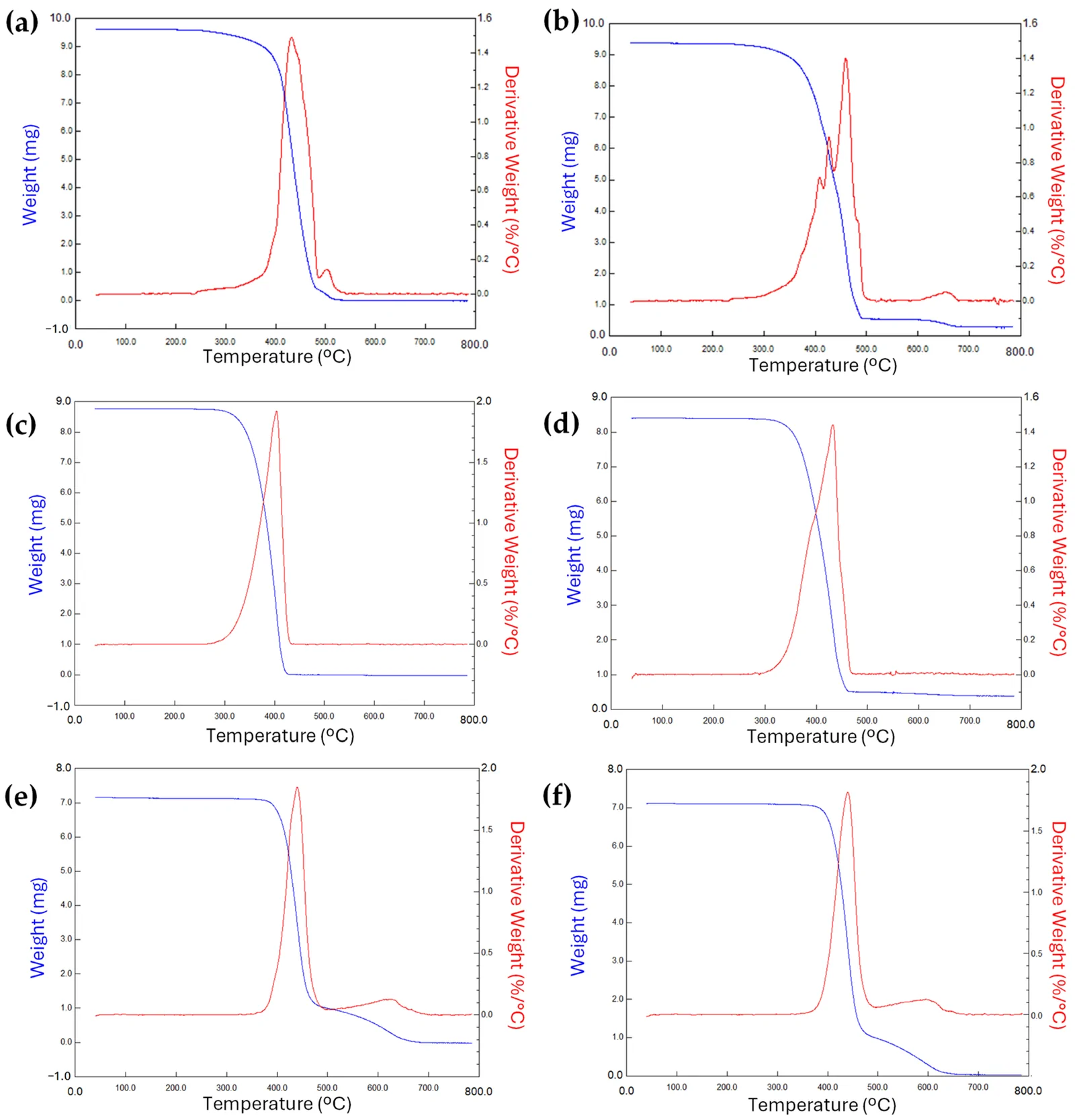
TGA curves of (**a**) PE; (**b**) RPE; (**c**) PP; (**d**) RPP; (**e**) PET; and (**f**) RPET.

**Figure 4:**
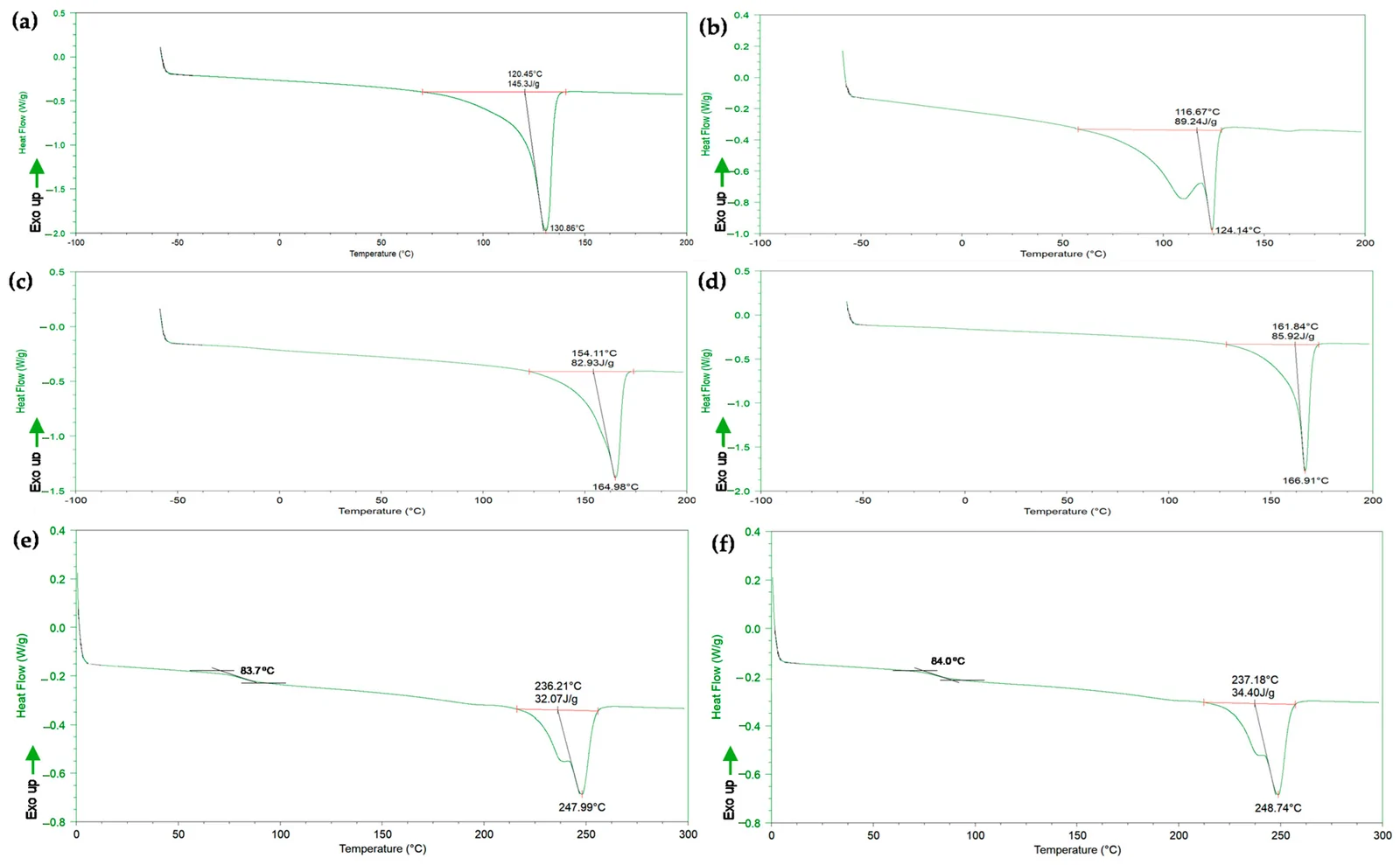
DSC curves of (**a**) PE; (**b**) RPE; (**c**) PP; (**d**) RPP; (**e**) PET; and (**f**) RPET.

Regarding the thermal properties, they were studied by TGA (Figure 3) and DSC (Figure 4). The material’s loss due to the increase in the temperature was analyzed as well as the remaining residue by TGA. In Table 2, the TGA results clearly show that PET and R-PET are very similar. Polyethylene (PE) was the material that showed the greatest difference between the virgin (Figure 3a) and recycled (Figure 3b) states. For starters, RPE (Figure 3b) showed multiple degradation peaks, which might not be the polymer degrading but may indicate the occurrence of overlapping degradation processes associated with fractions of different thermal stability or less thermally stable additives, highly branched chains or degraded/oxidized polyethylene chains within the RPE [29]. In polypropylene (PP), the degradation temperature was higher in the recycled polymer; this may be due to the different fibers, additives and crosslinking created, making it the mobility more restricted due to chain restrictions [30]. Finally, PP (Figure 3c) exhibits a single-step degradation because it is a purely hydrocarbon molecular backbone that undergoes uniform random chain scission. RPP (Figure 3d) also showed a single-step degradation with a somewhat broadened DTG peak. In contrast, PET contains rigid aromatic rings, and showed two degradation steps and two maximum degradation temperatures, evident in both PET (Figure 3e) and RPET (Figure 3f). The first typical of PET and the second one may be associated with the degradation of thermally stable residues and/or additional additives present in the materials. Similarly, PE can contain linear aspects (i.e., HDPE) and branching aspects (i.e., LDPE).

The results obtained from DSC thermograms (Figure 4) allowed us to obtain T_g_, T_m_ and the enthalpy of melting (Table 3).

The enthalpy shows a great change in the polyethylene (RPE, Figure 4b); this could be due to the increase in impurities, reducing the melting by hindering the crystallinity and therefore the melting point. In this line, two peaks are shown in the melting, referring to two different crystal populations with different melting points in RPE. In the same line as TGA, this could be due to the presence of highly branched chains, impurities, additives, etc. PP showed the typical melting peak at around 164 °C (Figure 4c) without significant changes in the RPP (Figure 4d), such as the appearance of new melting peaks. Two peaks are also shown in PET (Figure 4e) as well as in RPET (Figure 4f), which can be explained by the annealing process leading to two species of crystallization. As for PP, recycling causes chain scission; this degradation typically lowers overall crystallinity and shifts melting/crystallization peaks, as previously observed for recycled PP containing additional polymeric species as contaminants [31]. The glass transition temperature could not be detected for PE and PP, while that of PET did not change significantly between recyclates and virgin materials. T_g_ is highly dependent on polymer structure and chain mobility. This suggests that the addition of different species in the recyclates does not substantially affect the segmental mobility of the polymer chains.

**Figure 5:**
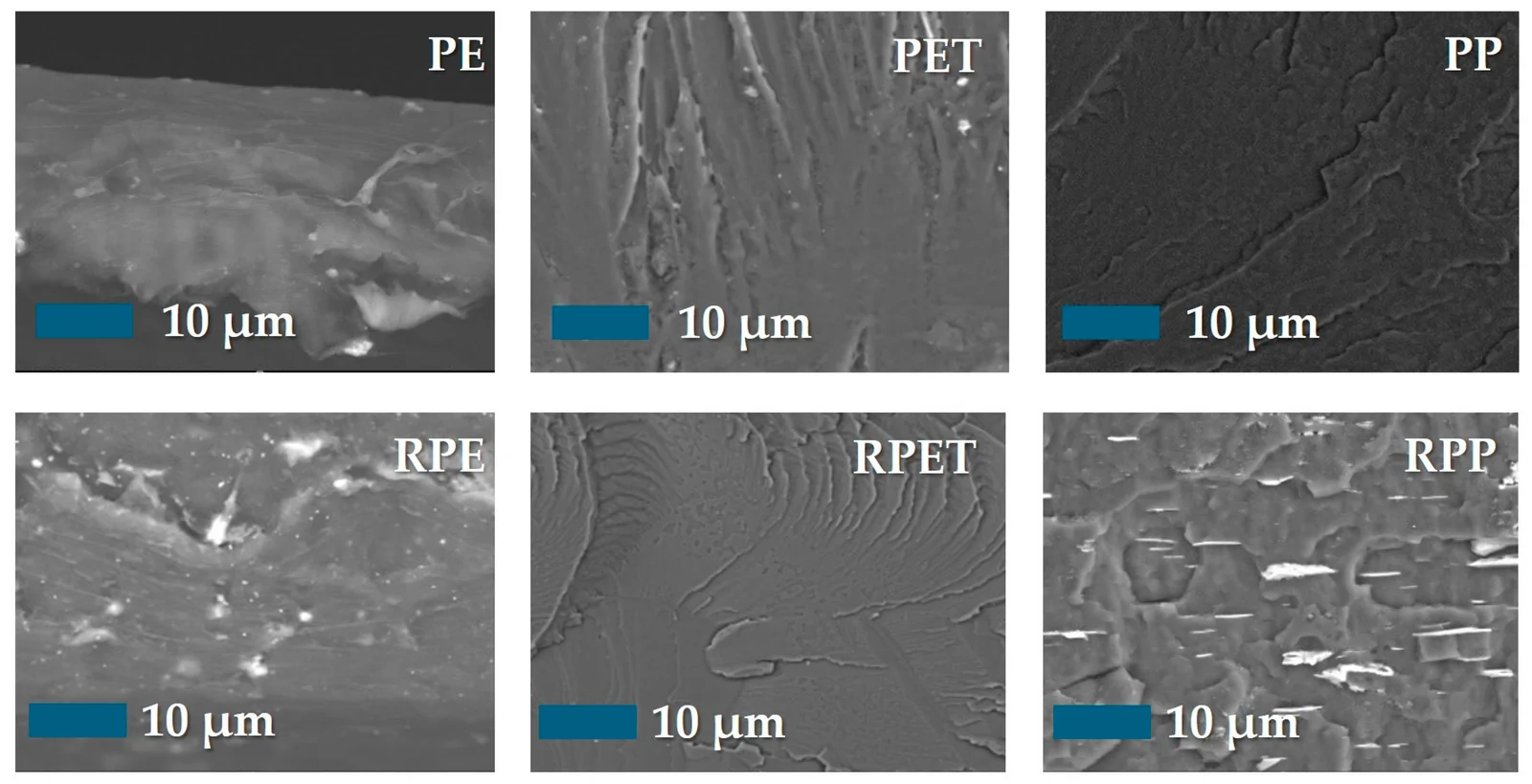
SEM images of virgin and recycled PE, PP and PET.

The morphology of the virgin and recycled materials was investigated by assessing the microstructure of their cryofracture surfaces (Figure 5).

EDX analysis revealed traces of different elements in PP and PE, such as Ca, Si, Ti, Al, Cu, K and Mg in RPE and Mg, Si, Al and Cl in RPP, whereas these elements were not detected in PET. These inorganic species may originate from additives, fillers, pigments or contaminants introduced during the previous service life and/or during the recycling process. However, their specific origin cannot be unequivocally established from the available EDX data. The presence of these additional inorganic species may influence the physical and mechanical properties of RPP and RPE compared with their virgin counterparts. Moreover, their presence could potentially affect not only further recycling processes but also the management of their waste at the end of the life cycle, leading to landfilling or incineration.

**Figure 6:**
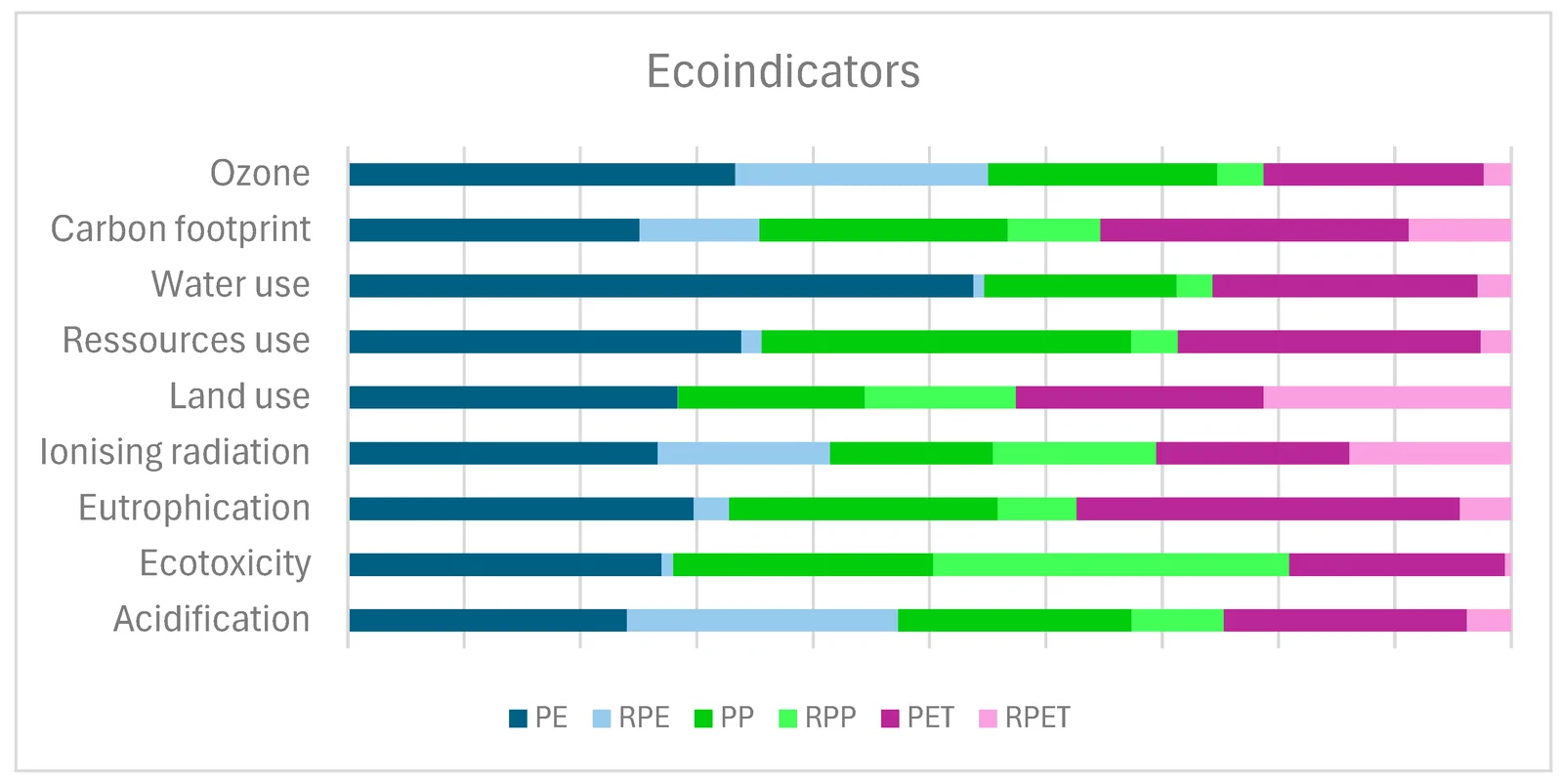
Bar diagram of the eco-indicators for virgin PET, PP, PE and recycled counterparts subjected to the study.

An LCA of all the materials subjected to this study was performed. As can be observed in Table 4 and Figure 6, when comparing the different recycled polymers, RPE shows the worst acidification and ozone. RPP shows great eco-indicators except for ecotoxicity, which is the highest. Even though RPET shows good results, the land use is too high. The three recycled polymers then show low eutrophication and high ionizing radiation. As for the virgin polymers, PE has the worst condition, with eco-indicators much higher than PET and PP. Thus, the better improvement was observed in RPE (especially in ecotoxicity, eutrophication and resources used). As expected, most of the eco-indicators improved when using recycled materials. RPP has been associated with the formation of micro- and nanoplastics because their chains have been weakened during the recycling process, making them keen to release contaminants to the environment [32].

## 4. Discussion

**Figure 7:**
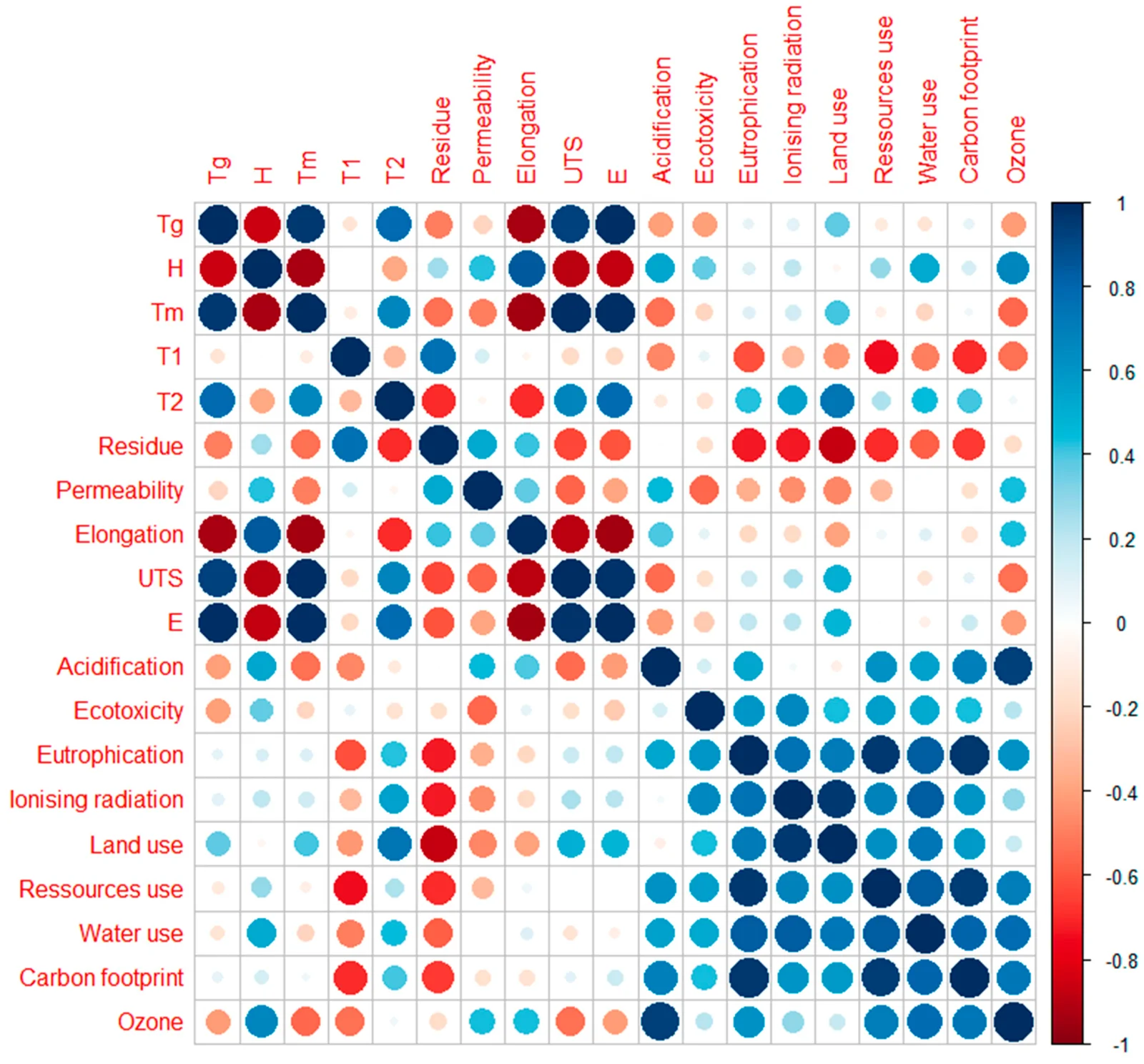
Diagram of correlation between physicochemical, thermal, water barrier and mechanical properties with the eco-indicators for all the polymers within the study.

A matrix correlation was performed, analyzing all the properties studied for the different polymers studied here and their eco-indicators, with the aim of finding links and relationships that help to understand the physics and chemistry of them all, is reported in Figure 7.

A trend was found suggesting a link between the thermal properties T_g_ and T_m_ and the ultimate tensile strength and the Young’s modulus, but it hinders the elongation at break. So, the greater melting temperatures and lower enthalpies result in more rigid materials. One reason may be the difference in crosslinking density, which increases the glass transition temperature by restricting the polymer chains [33,34]. Higher crosslinking makes molecules stiffer and more compact, but more difficult to disentangle, leading to lower elongation. As for permeability, the trend shows that the greater it is, the greater the elongation and the lower the UTS and E values; this trend means that higher polymer chain mobility may favor both molecular transport and deformation.

Another interesting trend was found between eco-indicators and the temperature decomposition peak measured by TGA, especially with the resources’ sources, eutrophication and carbon footprint. The link is negative, so the higher the temperature, the more the materials need to decompose; this could be related to a lower carbon footprint, since the material would be more thermally stable. However, this relationship should not be directly interpreted, since carbon footprint is determined primarily by the production and life-cycle inventory of each material rather than by its thermal stability. Except for ecotoxicity, land use and ionizing radiation, the rest of the eco-indicators show apparent trends of direct association. The acidification refers to the segregation of acidifying species coming from the material, but there is a trend that could relate it to permeability, as more channels facilitate diffusionr [34].

## 5. Conclusions

From a physico-chemical point of view, PET, assessed by ATR-FTIR, showed the same vibrational functional groups in recycled versus virgin materials. This agrees with the results of the SEM images, as both topologies were the same. TGA showed mainly one curve, showing a homogeneous decomposition in both samples. On the contrary, PE and PP showed morphological heterogeneities in the SEM images, while revealing the presence of additional inorganic traces measured by EDX analysis, not detected in their virgin counterparts.

The UTS (ultimate tensile strength) and Young’s modulus go from the highest to the lowest for PET, PP and PE, in contrast to the elongation at the break. Although slight differences in mechanical properties were observed between the virgin and the recyclated, the recycled materials retained mechanical performance comparable to that of their virgin counterparts, and it can be thought that the potential presence of additives such as fillers achieved the target mechanical properties.

The WVTR is similar except for PP, with RPP showing a much higher value than virgin PP. This difference between virgin and recyclates may reflect that reprocessing causes chain scission that could lead to increased polymer chain mobility, changing the water permeability.

The carbon footprint, eutrophication, ozone, water use and most of the eco-indicators were much higher in the virgin polymers than in the recyclates, which makes recycling a suitable sustainable option for plastics.

As for the technical properties, there are slight differences between PE and PP and their recyclates counterparts; in PET, however, all the properties were very similar. All these slight differences make them still suitable for the market. Nevertheless, the presence of additional inorganic species detected in RPE and RPP by the EDX in SEM, together with the differences observed by ATR-FTIR and in TGA, highlights the greater compositional complexity of these recycled materials compared with their virgin counterparts. These differences may arise from additives, fillers, pigments, contaminants, or degradation products associated with their previous use and recycling history. Their potential influence on the functional properties and further recyclability of these materials should therefore be considered.

## Figures and Tables

**Table 1:** Water vapor transmission rate of virgin PET, PP, PE and recycled counterparts.

Sample	WVTR (g/Day m^2^) ^1^	Standard Deviation
PET	2.99	0.87
RPET	3.10	0.31
PP	10.59	1.73
RPP	23.90	1.83
PE	0.08	0.03
RPE	0.34	0.08

^1^ WVTR values are normalized to 100 µm.

**Table 2:** TGA results of virgin PET, PP, PE and recycled counterparts.

Sample	T_1_ (°C)	T_2_ (°C)
PE	436.0	502.4
RPE	451.0 (412.0, 430.0, 483.0)	642.0
PP	425.0	-
RPP	471.0	-
PET	439.9	617.8
RPET	442.9	607.7

**Table 3:** DSC results of virgin PET, PP, PE and recycled counterparts.

Sample	T_g_ (°C)	T_m_ (°C)	AH_m_	AH°_m_	Number of Peaks
PE	-	130.9	145.3	290	1
RPE	-	121.3	103.7		2
PP		165.0	82.9	207	1
RPP		166.9	85.9		1
PET	83.7	248.0	32.1	140	2
RPET	84.0	248.7	34.4		2

**Table 4:** List of eco-indicators for virgin PET, PP, PE and recycled counterparts subjected to the study.

	Acidification	Ecotoxicity	Eutrophication	Ionizing Radiation	Land Use	Resources Use	Water Use	Carbon Footprint	Ozone
PE	0.006	0.697	0.015	0.191	3.642	75.766	1.562	2.091	0.008
RPE	0.005	0.027	0.002	0.106	0.009	3.970	0.027	0.860	0.005
PP	0.005	0.577	0.012	0.100	2.061	71.077	0.480	1.780	0.004
RPP	0.002	0.790	0.003	0.100	1.667	9.006	0.089	0.665	0.001
PET	0.005	0.479	0.017	0.119	2.737	58.254	0.662	2.209	0.004
RPET	0.001	0.014	0.002	0.100	2.733	5.904	0.083	0.733	0.001

## Data Availability

The data presented in this study are openly available in the e-cienciaDatos repository (Consorcio Madroño) at https://doi.org/10.21950/ZM8YW9.

## Abbreviations

The following abbreviations are used in this manuscript:

CE	Circular economy
PE	Polyethylene
PP	Polypropylene
PET	Polyethylene terephthalate
PPWR	Packaging and Packaging Waste Regulation
HDPE	High-density polyethylene
LDPE	Low-density polyethylene
GHE	Greenhouse gas emission
ATR-FTIR	Attenuated Total Reflectance Fourier Transform Infrared
SEM	Scanning electron microscopy
WVTR	Water vapor transmission rate
LCA	Life cycle assessment
TGA	Thermogravimetric analysis
DSC	Differential scanning calorimetry
T_g_	Glass transition temperature
T_m_	Melting temperature
UTS	Ultimate tensile strength
E	Young’s Modulus
H	Enthalpy
T1	First maximum degradation temperature in TGA
T2	Second maximum degradation temperature in TGA
